# Inclusion through technology: findings from a public engagement approach

**DOI:** 10.3389/ijph.2026.1608949

**Published:** 2026-07-08

**Authors:** Barbara Catherine Wortmann, Iris de Boer, Vanessa Grand, Heiner Baur, Anja Maria Raab

**Affiliations:** 1 Division of Physiotherapy, School of Health Professions at Bern University of Applied Sciences, Bern, Switzerland; 2 OMNIA Health Services AG, Oberwil, Switzerland; 3 Physiotherapy Department, Reha Rheinfelden, Rheinfelden, Switzerland; 4 Institute for Collaborative Practice and Leadership in Healthcare, School of Health Professions at Bern University of Applied Sciences, Bern, Switzerland

**Keywords:** assistive technology, disability, inclusion, participation, public engagement

## Abstract

**Objectives:**

Many people with disabilities in Switzerland face exclusion, especially in mobility, work, and leisure. Assistive technologies can support participation and wellbeing, particularly when developed with user involvement. This study explored societal perspectives on inclusion through technology using a public engagement approach.

**Methods:**

The study took place at the Swiss Abilities Fair 2024 in Lucerne. At an interactive booth, visitors tested assistive technologies like a robotic arm and a recumbent trike. They shared their views in semi-structured interviews conducted by physiotherapists and a wheelchair-using researcher. Interviews were recorded, transcribed, and analyzed using qualitative content analysis. The sample included people with and without disabilities.

**Results:**

Forty-one people participated. Non-disabled participants emphasized equality and acceptance, while those with mobility impairments focused on barriers and support. Assistive technologies were seen as key to independence, though gaps in innovation, access, and affordability remain.

**Conclusion:**

Inclusion is a shared societal responsibility that requires awareness, participation, and adaptable solutions. While assistive technologies support independence and participation, interviewees emphasized that true inclusion also depends on affordability, access, user involvement, and broader structural change.

## Introduction

In recent years, inclusion has gained increased attention in social and political discourse as a core principle of a just and diverse society. It is described as a universal human rights principle that enables self-determined living and equal participation in society [1]. “Aktion Mensch”- a German non-profit organization advocating for the rights and inclusion of people with disabilities - offers a more accessible definition: ‘Inclusion means everyone gets to participate—regardless of what you look like, what language you speak, or whether you have a disability’ [2].

An inclusive society means access not only to leisure, but also to family life, education, adequate living standards, and social protection. In Switzerland, around 1.8 million people live with disabilities [3]. According to the Inclusion Index by Pro Infirmis - a Swiss organization providing services and advocacy for people with disabilities - four out of five individuals report feeling excluded, especially in mobility, employment, politics, and leisure [4]. Switzerland still has considerable room for improvement in promoting inclusion [5].

Disabilities can limit participation in daily life and therefore, inclusive structures are essential for equal opportunities. Alongside organizational and communicative strategies, assistive technologies are key to realizing this human right by supporting functioning, enhancing health, wellbeing, and participation. According to the World Health Organization (WHO), over 2.5 billion people worldwide need assistive products, a number expected to rise to 3.5 billion by 2050 due to global aging. Some need them temporarily, others long-term or lifelong.

Previous research consistently shows that assistive technologies can significantly improve autonomy, quality of life and participation in social and economic life when they are appropriately designed and implemented. Studies highlight their role in enabling communication, mobility, learning and workplace participation for people with disabilities [6].

Access is often hindered by limited financial ressources, narrow product ranges, procurement issues, weak policies and sociodemographic barriers [7]. In addition, research indicates that technological solutions alone are often insufficient to achieve meaningful inclusion, as social environments, stigma and institutional barriers strongly influence whether technologies can support participation [8].

The WHO recommends involving users and caregivers in development of assistive technologies to ensure safety, effectiveness, and affordability. Public awareness and stigma reduction are also essential for inclusion and innovation [7].

User-centered design should guide research to ensure technologies meet real needs. Recent studies therefore emphasize participatory approaches and the importance of understanding how technologies are perceived in everyday social contexts. However, existing research has largely focused on technological development, clinical outcomes or specific institutional settings such as schools or workplaces, while broader societal perspectives on assistive technologies and inclusion remain comparatively underexplored [9].

Public engagement, understood as dialogue and collaboration between science, society, and stakeholders, can increase acceptance, awareness, and public investment. Unlike traditional science communication, it emphasizes interaction and co-creation, making research more transparent and responsive to societal concerns [10].

In light of the aforementioned aspects, the present study explored societal perspectives on inclusion through technology using a public engagement approach understood as a dialogical and participatory method rather than a theoretical framework. By engaging visitors of an assistive technology exhibition through hands-on interaction and short questions, the study sought to capture diverse understandings of inclusion, perceived barriers and expectations toward assistive technologies in an everyday public context for the target group.

## Methods

### Design

This exploratory qualitative study was based on short, open-ended interviews aimed at capturing diverse participant perspectives and experiences on the topic of “inclusion through technology”.

### Setting

The study was conducted at the Swiss Abilities Fair 2024 in Lucerne, a national event promoting inclusion. The fair addresses physical, intellectual, psychological, and sensory disabilities. Exhibitors present assistive technologies, offer consultations, and hands-on experiences. Through exchange and interaction, the fair fosters inclusion and empowerment [11].

**FIGURE 1:**
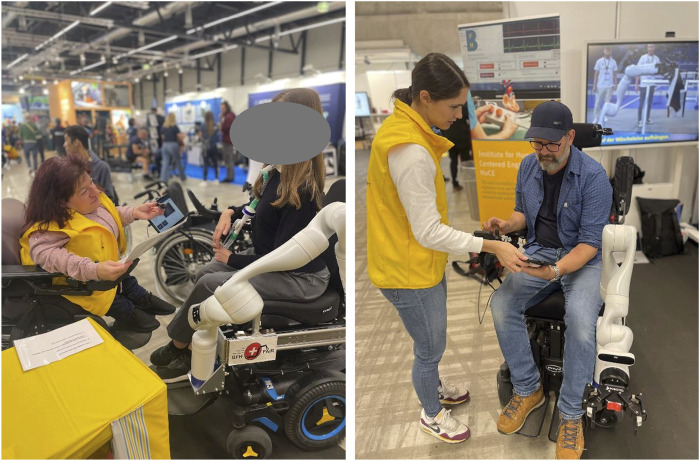
An interactive booth at the Swiss Abilities Fair 2024 in Lucerne, where visitors tested a robotic arm designed to support individuals with limited arm function (Switzerland, 2025).

The core of the study was an interactive booth with visitors tested a robotic arm developed for people with limited arm function (Figure 1) and a recumbent trike. Guided by a researcher, participants used the arm from a power wheelchair to hand an apple to a team member, who then read aloud a number corresponding to a predefined question card on inclusion and technology. These interactions formed the basis for short conversations, during which participants answered two open-ended questions and could pose two of their own, initiating dialogue. The hands-on experience encouraged perspective-taking and deeper engagement with the topic.

### Data collection

During the two-day Swiss Abilities Fair, short conversations were conducted by three physiotherapists and a wheelchair-using researcher. The team’s diverse backgrounds contributed to both professional and personal perspectives. Each short interview began with the question: *“What does inclusion mean to you?”* followed by a participant question on inclusion and technology, one from the researcher, and a final participant question. The sequence was flexibly adapted. To ensure that participants could speak freely the short interviews were conducted at a designated area of the booth.

Participants interested in inclusion through technology were invited to continue the conversation at a nearby table with a researcher (wheelchair user or non-disabled team member) allowing for a quieter setting and greater privacy. These in-depth interviews began with the same initial question and covered sociodemographic data, understanding of inclusion, societal inclusion, and the role and cost of assistive technologies.

Before participation individuals were informed about the study aim, procedure, voluntary nature of participation and their right to withdraw at any time without consequences. Oral informed consent was obtained. No personal information was collected and the data was anonymized during transcription and analysis. This flexible questioning approach is consistent with exploratory qualitative research where the aim is to capture diverse perspectives and everyday understandings rather than to conduct in-depth narrative interviews.

The interview guide (Supplementary Material 1) was developed through a multi-stage, iterative process. Two team-members drafted seven thematic blocks based on literature and clinical experience, refined in a plenary session with four others to ensure scientific and practical relevance. For validation, two external health researchers reviewed the guide; their feedback was incorporated following qualitative research criteria such as clarity, openness, and relevance. The final guide was printed for standardized use during extended interviews. Short question cards (Supplementary Material 2) reflected key questions from the full guide. Short interviews, although brief, were designed to capture focused perspectives on a single open-ended question and a definition within the public engagement setting. These contributions provided concise, experience-based insights that complemented the more in-depth interviews.

All interviews were recorded using two DJI Mic2 microphones and iPhones.

### Sampling

Participants included visitors and exhibitors at the Swiss Abilities Fair who showed interest in inclusion and assistive technologies. Recruitment was spontaneous. Eligible were individuals aged 18 or older, without cognitive impairments, and fluent in German, French, or English. To capture a range of perspectives, the sample included people with and without mobility disabilities. Visitors who showed interest in the topic or the interactive setup were invited to participate. Due to the open public setting the number of individuals approached and those who declined participation were not systematically recorded.

### Transcription

All interviews and short conversations were audio-recorded and transcribed. Files were coded and anonymized. Transcriptions were conducted according to established coding procedures [12] using MAXQDA or Töggl for Swiss German dialects.

Five physiotherapists involved in developing the interview guide transcribed the material, ensuring contextual accuracy. A training session ensured consistent application of the rules. All transcripts were reviewed by two researchers; unclear passages were discussed and revised collaboratively.

### Data analysis

After transcription, responses were organized in a table aligning each question with corresponding answers to facilitate comparison. Based on this structure, the RADaR (Rigorius and Accelerated Data Reduction) technique [13] was applied to iteratively reduce the data and extract key statements. Each response was assigned one or more categories reflecting its core message, enabling thematic condensation. Main categories were identified to represent different perspectives. Data coding was conducted by two physiotherapists who were members of the research team and who conducted the interviews and the fair. To enhance reliability, coding was conducted independently and then consolidated. Following a combined deductive-inductive approach [14], the data were systematically analyzed to identify key themes and patterns. Given the exploratory and time-bound nature of the setting data collection was not guided by a predefined criterion. Instead the saturation was assessed retrospectively based on the recurrence of the key themes across interviews and short conversations.

## Results

A total of 42 interviews were conducted; 26 in-depth and 16 short conversations, lasting 4 h and 55 min. In-depth interviews lasted between 5:31 to 17:38 min; short ones ranged from 54 s to 5:18 min. One short interview was documented with written notes only, as the participant declined audio recording. One interview was excluded due to thematic inconsistencies. The participant’s responses diverged from the study focus and did not permit meaningful interpretation.

Among the 42 participants, sex distribution was fairly balanced, while age differences were notable, most participants without mobility impairments were aged 31–60, whereas nearly half of those with disabilities were 18–30. Participants were categorized based on self-reported mobility impairment. No systematic data was collected on other types of disabilities (Table 1).

**TABLE 1:** Participant characteristics (Switzerland, 2025).

Variable	Category	Participants without mobility impairments n = 27 (%)	Participants with mobility impairments n = 15 (%)
Sex	Male	12 (44%)	6 (40%)
​	Female	15 (56%)	9 (60%)
Age in years	18–30	0	7 (47%)
​	31–60	22 (81%)	4 (27%)
​	61+	2 (7%)	3 (20%)
​	Unclear	3 (11%)	1 (7%)

The interviews revealed clear differences between affected and non-affected individuals. Divergences emerged in understandings of inclusion and societal barriers, perceptions of assistive technologies, their costs and funding, and their role in fostering independence and inclusion.

The analysis revealed both convergent and divergent perspectives between participants with and without mobility impairment across several themes.

The themes summarized in Table 2 are described in more detail in the following sections.

**TABLE 2:** Overview of convergent and divergent perspectives between participants with and without mobility impairment (Switzerland, 2025).

Theme	Participants with mobility impairment	Participants without mobility impairment	Convergent/Divergent
Understanding of inclusion	Experience-based, focused on autonomy and self-determination	Abstract, normative understanding of inclusion	Divergent
Perceived social barriers	Emphasis on everyday physical and social barriers	Awareness of barriers, less detail	Divergent
Role of assistive technologies	Central for independence and daily functioning	Viewed as supportive and beneficial	Convergent
Access and affordability	High relevance and concern	Recognition of cost-related barriers	Convergent
Awareness of diverse impairment	Frequent emphasis on non-mobility impairment	Less frequently mentioned	Divergent

In the following section, labels such as “Supplementary Material 3-2” indicate the corresponding numbered quotations listed in Supplementary Material 3. A visual summary of all key study findings is provided in Supplementary Material 4, which presents the results in a comprehensive poster format.

### Understanding of inclusion and societal barriers

The analysis revealed that individuals’ views on inclusion are shaped by their lived realities. Clear differences appeared: participants with mobility impairments saw inclusion as a concrete, experience-based concept, while others viewed it as an abstract societal ideal.

“To me, inclusion means that people with disabilities can participate in social life as fully and with as few restrictions as possible.”

Male participant without mobility impairment, age mid-50s

Affected individuals particularly emphasized the importance of self-determination, autonomy, and the removal of physical and social barriers (Supplementary Material 3-2).

Only a few interviewees saw inclusion as extending beyond physical impairments (Supplementary Material 3-3).

Despite the Swiss Abilities fair’s focus on self-determined living, eight participants could not define inclusion.

Regardless of perspective, several barriers persist, especially in public spaces, where issues like non-level bus stops and inaccessible buildings remain (Supplementary Materials 3-5, 3-7).

“Sometimes it says ‘wheelchair accessible,’ but when you get there, it does not actually work, maybe the wheelchair is too heavy or too wide, or there’s a step after all.”

Female participant with mobility impairment, age unknown

A recurring theme was frustration over inaccessible public transport, especially the lack of options for spontaneous travel (Supplementary Material 3-9).

“A wheelchair user has to carefully choose where to spend their vacation. Full inclusion would mean being able to travel freely and decide spontaneously where to stay, but that’s definitely not possible.”

Male participant without mobility impairment, age 48

Some infrastructures are labeled accessible but fail to meet the real needs of people with mobility impairments (Supplementary Material 3-10).

“There are many obstacles that wheelchair users simply cannot overcome. Sometimes a place is labeled ‘wheelchair accessible,' but when you arrive, it turns out not to be (…) These are the kinds of barriers that stop me.”

Female participant with mobility impairment, age unknown

Interviews showed that blind or visually impaired people often feel excluded from public inclusion debates, which in Switzerland tend to focus on wheelchair users (Supplementary Materials 3-12–3-14).

Inclusion remains poorly understood, with limited awareness of diverse impairments in public services.

“Bus stops may be inclusive in theory, but for example, they do not have automatic ramps. The driver has to come out and unfold it manually, and everyone gets annoyed because it causes delays. That’s exactly the issue: the problems are caused by the people behind the system.”

Female participant with a mobility impairment, age approx. 20

Societal acceptance remains a challenge. Prejudices persist, and many report subtle discrimination, like not being taken seriously or being addressed indirectly. A lack of sensitivity can also lead to intrusive help, such as pushing a wheelchair without consent.

“Or they’re just grabbed by the wheelchair and pushed somewhere.” Male participant without mobility impairment, age mid-50s

Building on the understandings of inclusion, the following section focuses on how participants perceived the role of assistive technologies in everyday life.

### Understanding of assistive technologies

Participants understood assistive technology differently. Some linked it to mobility aids like wheelchairs or lifts, others to digital tools like voice control or apps. Sometimes, it was broadly described as ‘technology that helps,’ without specific examples (Supplementary Material 3-17).

“It can range from a walking stick to a wheelchair, or even a car adapted for wheelchair users. Possibly glasses as well.”

Male participant without mobility impairment, age 40

Not all participants knew the term or equated it with familiar everyday aids.

“To be honest, I cannot really picture much in that regard. Maybe just that (…) technology helps and supports people who need something.”

Female participant with a mobility impairment, age 27

### The role of assistive technologies in promoting independence and inclusion

Assistive technologies that support independence may help counter societal insensitivity, according to both disabled and non-disabled individuals. Mobility aids like wheelchairs enable users, especially those with severe paralysis, to manage daily life autonomously. One participant, for instance, steers his wheelchair with his chin and uses voice control on his smartphone to stay largely independent (Supplementary Material 3-21).

“I see this very positively. This is a part of technology developed for people with disabilities. When I look around today and see how many people are walking with prostheses, without them, many more would be in wheelchairs.”

Female participant with no mobility impairment, age 50

Significant gaps remain, as inclusion has not progressed equally across all life areas. Some participants stressed the value of access-enhancing technologies, like faster lifts or automatic doors, that can greatly improve daily autonomy.

“What I notice is that there’s still very little available for people who are blind. It’s basically just a cane. (…) Maybe something could be done with sensors… Yes. Nowadays, there are so many sensors that can detect everything, like when you reverse a car.”

Male participant with no mobility impairment, age 75

Many people with disabilities report not only a lack of assistive technologies but also limited access to key information, such as on accessible spaces, available services, legal rights, and entitlements (Supplementary Material 3-23, 3-25).

“From my point of view, people who are affected should simply receive more information, about what support they are entitled to, what they can benefit from, or where they can turn to for help.” Female participant with no mobility impairment, age 52

In leisure and sports, participants noted clear gaps in inclusive offerings. Clubs and activities were often poorly adapted—due to structural barriers and limited staff awareness. Lack of know-how and hesitation were common barriers (Supplementary Material 3-27).

“I notice that the barriers are still quite high in this area. It’s often quite difficult. (…) The people involved, like trainers, often lack the necessary know-how. They may be afraid of not being able to implement things properly, or they simply do not have the means to adapt the conditions (…) There may also be certain prejudices toward people with disabilities.”

Male participant with no mobility impairment, age 51

A recurring theme was the lack of human and financial resources in schools, contributing to ongoing shortcomings in disability inclusion (Supplementary Material 3-29).

Our daughter has CP and attended a public school from kindergarten through second grade. Then the teachers started to resist. (…) We felt it was no longer working, even with the support of an assistant.”

Female participant with no mobility impairment, age 52

Due to structural shortcomings, children are often excluded from inclusive early education and placed in special schools.

“Children are sent to special schools or schools for children with special needs in another village. And then they’re just gone from the community. They’re no longer really part of it. I think that’s such a shame. We should start with the youngest children. (…) I mean, my own children have never had any contact with children with disabilities.”

Female participant with no mobility impairment, age 44

In contrast, simple structural measures are often implemented (Supplementary Material 3-31). Barriers may also emerge later in the careers of people with disabilities (Supplementary Material 3-32).

Overall, experiences show that, beyond removing structural barriers, greater societal awareness of disabled people’s abilities is crucial.

While assistive technologies were widely viewed as enabling autonomy, participants also highlighted practical challenges related to access and affordability, which are addressed in the following section.

### Costs and funding of assistive technologies

Participants called for shared funding of workplace adjustments between employers and insurers and expressed openness to remote work (Supplementary Material 3-33).

“I think we should aim for a kind of 50–50 solution, where half of the costs are covered by the insurance and the other half by the employer.”

Male participant with mobility impairment, age 69

Assistive technologies are key to improving quality of life and independence for people with disabilities (Supplementary Material 3-35).

“Very much so, it’s definitely something valuable. I’m grateful for the wheelchair, I’m grateful for the orthoses. Without them, I would not be able to participate in everyday life the way I do now.”

Female participant with mobility impairment, age 27

The need to further develop assistive technologies was also emphasized.

“I think it’s very important. In my view, assistive devices should be used to solve as many situations as possible, but they also need to be easy to operate, especially through voice control. I say this because I’m so highly paralyzed that I cannot easily use my hands for anything.”

Male participant with mobility impairment, age 69

High acquisition costs and unclear cost coverage were frequently identified as major challenges (Supplementary Material 3-39).

“I think there should always be a solid framework, but the question is whether you have to pay for it yourself or not. As people with disabilities, we usually do not have the financial means to cover these costs ourselves.”

Female participant with mobility impairment, age 22

Interviews revealed shared views among participants with and without disabilities on the need for fairer cost distribution of assistive devices (Supplementary Materials 3-41–3-45).

“In general, I think it’s a good idea, similar to health insurance, if users also contribute a share. But it has to be adjusted to their financial situation.”

Male participant without mobility impairment, mid-40s

Assistive technologies are vital for people with disabilities and can also benefit society by improving the accessibility and usability of public and everyday environments for all.

“An automatic door helps not only wheelchair users, it also benefits pedestrians or waiters. (…) I noticed it myself when I had a stroller. Now you can just roll in. These small things serve everyone.”

Female participant without mobility impairment, age 50

## Discussion

This study aimed to capture and critically reflect on societal perspectives on inclusion through technological support using a public engagement approach. The findings indicate that while promoting inclusion remains a significant challenge on both technological and societal levels, it also presents opportunities to enhance the quality of life and participation of people with diverse impairments through targeted measures.

While existing research has examined inclusion from legal, clinical and policy perspectives, fever studies have explored how inclusion is understood and negotiated with the general public, particularly in relation to emerging assistive technologies. The literature often focuses either on technological innovation or on social barriers but rarely integrates both perspectives through direct public engagement. As a result, there remains limited empirical insight into how societal attitudes, lived experiences and technological possibilities intersect in shaping inclusion in everyday contexts [15–17].

### Understanding of inclusion and societal barriers

People with and without disabilities often understand inclusion differently. While individuals without disabilities describe it in broad societal terms, those with disabilities view it more personally as autonomy and control over their own lives. This divergence is reflected in the literature, which shows that inclusion is interpreted depending on the framework (e.g., legal, educational, or social policy) and varies across cultural and social contexts [18].

Public perceptions of accessibility also reveal important gaps. Accessibility is often narrowly associated with physical infrastructure, such as ramps or elevators. However, people with sensory disabilities, such as blindness, frequently feel overlooked. This limited view fails to reflect the diverse ways people navigate public spaces. Chidiac et al. emphasize that current standards often ignore sensory barriers and call for more inclusive design that considers diverse needs and assistive technologies [19].

Interviewees also reported frequent experiences of prejudice in educational and employment settings, where they often do not feel treated as equals. These findings align with research showing that decisions like reduced school timetables or denied employment disproportionately affect certain disabilities [20]. Such practices reflect persistent hierarchies of acceptability, even in settings that claim to be inclusive. Ableism refers to discrimination and negative attitudes toward people with disabilities ranging from exclusion to subtle stereotypes. Ableist attitudes have been shown to vary by disability type and social context [20]. Importantly, people with personal or close experience of disability tend to express fewer ableist views, while limited contact is linked to more exclusionary attitudes. This suggests that everyday inclusion may help reduce prejudice.

Our findings also highlight substantial barriers in daily life, especially in politics, employment, and mobility. Many interviewees reported difficulties using public transport due to the need for advance planning, which limits autonomy. Although accessibility is legally established, implementation often falls short. Digital infrastructures, such as websites, are also frequently inaccessible. These observations align with the *Inclusion Index 2023*, which found that 80% of respondents with disabilities face major limitations in at least one life domain, and nearly one-third report challenges with public transport [4].

While prior research acknowledges that definitions of inclusion vary across contexts, it rarely captures how these differing understandings coexist and potentially conflict within everyday societal interactions [21]. Our findings extend this by demonstrating how these divergent perspectives shape expectations, experiences and perceived barriers in daily life.

### Understanding of assistive technologies

Interview data revealed varied interpretations of assistive technology, ranging from traditional mobility aids to digital tools like voice control and robotics. Despite these differences, its importance for autonomy and participation was consistently acknowledged. Assistive technologies not only support people with disabilities but also benefit society, for example, through automatic doors that assist parents with strollers or older adults using walkers. Their development should be seen as a societal investment.

These findings align with recent literature showing that public understanding of assistive technologies is shaped by personal relevance, exposure, and broader societal narratives [22]. Autonomy is described as a complex and sometimes paradoxical concept, especially in health and disability contexts, where technology can both enable and constrain agency [23]. This shows that assistive technologies are not merely tools but embedded in social, cultural, and institutional contexts. Enhancing public understanding is key to fostering inclusive attitudes and informed policymaking.

### The role of assistive technologies in promoting independence and inclusion

Our findings show that assistive technologies are essential to autonomy and inclusion. Participants described how tools like powered wheelchairs, voice-controlled smartphones, and automatic doors support independent living and access to public spaces. These technologies were viewed positively by both disabled and non-disabled individuals, who also noted their broader societal benefits. However, barriers remain, and participants reported a lack of information and resources in education and leisure. Staff in schools and recreational settings were often seen as undertrained or unaware of inclusive practices. These insights are supported by a 2022 review showing that assistive technologies enhance inclusion in education, though gaps in teacher training remain [22]. A 2019 review on higher education similarly found improvements in academic outcomes and psychosocial wellbeing [24].

The WHO emphasizes that assistive technologies are not merely supportive tools but essential enablers of rights, participation, and dignity. They are critical across all life domains, from education and employment to self-care and social engagement, and central to achieving the Sustainable Development Goals (SDGs) [7]. However, the WHO and UNICEF *Global Report on Assistive Technology* (2022) reveal considerable global disparities: in some low-income countries, only 3% of people have access to needed assistive products, compared to 90% in high-income countries. For instance, of the 80 million people worldwide needing a wheelchair, only 5%–35% have access, depending on location. Similarly, while 1.5 billion people experience hearing loss, current hearing aid production meets less than 10% of global demand [25].

While the benefits of assistive technologies are well documented, research often treats them as isolated interventions. Recent work highlights that assistive technologies are embedded within complex interactions between individual, social and environmental factors [26]. Our findings support that their effectiveness is closely tied to environmental, institutional and societal conditions suggesting that technological innovation alone is insufficient without parallel structural and cultural change.

### Costs and funding of assistive technologies

The study highlights the persistent challenge of high costs for assistive technologies and workplace accommodations. Participants emphasized the need for equitable cost-sharing between users, insurers, employers, and the state. This reflects international recommendations which calls for affordability, cross-sector integration and public awareness to improve accessibility and participation [25]. Systematic analyses further show that financial constraints, limited knowledge and broader systemic barriers remain key challenges for the development, accessibility and widespread adoption of assistive technologies [27].

Remote work emerged as a cost-effective strategy to reduce financial burden. The WHO and ILO emphasize that well-designed telework arrangements promote health, autonomy, and inclusion for people with disabilities [25, 28]. The OECD identifies remote work as key to inclusive labor market strategies, helping individuals with reduced work capacity stay employed [29]. These insights suggest that combining assistive technologies with flexible work policies is essential for equal access and long-term participation.

Across the thematic findings several cross-cutting insights emerge. Autonomy appears as a recurring concern shaping how conclusion, assistive technology and participation are understood across participant groups. While assistive technologies are widely perceived as enabling independence their potential is frequently constrained by persistent structural, organizational and financial barriers. In addition participants consistently emphasized that technological solutions alone is insufficient without broader societal awareness, inclusive attitudes and supportive institutional frameworks. Taken together, these observations highlight a critical gap in existing research: inclusion through technology cannot be fully understood without considering the dynamic interaction between technological innovation, societal attitudes and structural conditions. While previous studies often address these dimensions separately, our results demonstrate their interdepence in real-world inclusion.

These insights align with existing literature that frames inclusion as a shared societal responsibility rather than an individual challenge and emphasize the role of assistive technologies as enablers of rights and participation rather than optional add-ons. Importantly, the public engagement setting of this study highlights how dialogue and hands-on interaction can make these systemic dimensions visible to a broader audience, potentially contributing to greater awareness, reduced stigma, and more inclusive technological development.

### Limitations

This study draws on qualitative data collected at the Swiss Abilities fair, offering insights into public perceptions of inclusion and assistive technology. However, several limitations should be considered. Visitors may have been more familiar with or positively inclined toward the topic than the general population, introducing selection bias. The spontaneous recruitment likely favored individuals with above-average interest, while more critical perspectives may have been underrepresented. Responses to socially sensitive topics such as inclusion may also have been shaped by social desirability. Prior experiences with assistive technologies were not systematically assessed and may have influenced participants’ perspectives. Although some participants referred to personal experiences during the interviews, this factor was not controlled for and should be considered when interpreting the findings. The presence of a researcher using a wheelchair could have further influenced answers. Finally, the categorization and interpretation of responses involved subjective judgment by the researchers, which may have introduced bias.

### Impact and future directions

To reduce societal prejudice, inclusion should begin early in the education system. Early awareness fosters long-term understanding and can be supported through training on diversity and inclusive practices. Involving people with disabilities in outreach and project development further strengthens inclusion. These recommendations align with the *Public Engagement Kodex*, which offers guidance for participatory project design. Interview data also emphasizes the need to integrate the perspectives of those directly affected [30].

Assistive technologies are key to autonomy and participation. Their broader benefits, such as improved access for older adults or parents with strollers, highlight their societal relevance. Development should focus on affordable, adaptable solutions, including open-source models. Public funding and private-sector collaboration can enhance innovation and access. These technologies should be integrated into mainstream infrastructure, not treated as add-ons.

Political commitment is crucial, particularly for improving public infrastructure. Ensuring lasting accessibility requires regular review and adaptation. Uncertainty about how to implement inclusion often leads to exclusion, driven by limited options and limited awareness. Targeted training and a proactive mindset can help close this gap.

By combining public engagement with qualitative inquiry in a real-word setting this study contributes to bridging the gap between abstract policy and technological discussions and the lived realities and perceptions of society.

## Data Availability

The data generated and/or analysed in this study are available on reasonable request from the corresponding author.
