# Supplementary material for: Inclusion through technology: findings from a public engagement approach
Source: Int J Public Health. 2026 Jul 8;71:1608949. doi: 10.3389/ijph.2026.1608949 (PMC13388228; doi:10.3389/ijph.2026.1608949)
Supplement: Supplementary file 1 [file Supplementaryfile1.docx]

**Supplement 1: Interview guide in German and English**

Semistrukturiertes Zwiegespräch zum Thema “Inklusion” - Swiss Ability Messe 2024

Einleitung und Allgemeines:

1. Begrüssung und Vorstellung als Moderatorin
2. Dank an teilnehmende Personen, dass sie sich für das Interview zur Verfügung stellen und sich Zeit nehmen
3. Ungefähre Dauer des Interviews bekanntgeben (ca. 10 Minuten)
4. Interview wird aufgenommen, nach Datenauswertung und Publikation werden diese wieder gelöscht
5. Evtl. Ziel vom Gespräch kurz erläutern?

Rechte kurz erläutern:

- Daten werden vertraulich behandelt, anonymisiert und nur im Kontext des Projekts verwendet
- Teilnahme ist freiwillig
- Abbruch ist jederzeit möglich

Gibt es noch Fragen?

**Start des Interviews: Aufnahme starten**

| Themen | Hauptfrage | Konkrete Nachfrage | Aufrechterhaltungsfragen |
| --- | --- | --- | --- |
| Vorstellung | Könnten Sie kurz erzählen, wie alt Sie sind und was Sie beruflich machen? | 1. Geschlecht 2. Beruf? 3. Alter? |  |
| Einstiegsfrage/  Standardfrage | 1. Was verstehen Sie unter Inklusion? 2. Was verstehen Sie unter Assistenztechnologien? | 1. Warum denken Sie ist Inklusion in unserer Gesellschaft wichtig? |  |
| Erfahrungen mit Inklusion | 1. Welche persönlichen Erfahrungen haben Sie mit Inklusion gemacht? 2. Wenn ja, können Sie uns von einer positiven Erfahrung erzählen? 3. Gab es auch herausfordernde oder weniger inklusive Erlebnisse? | 1. Wie haben Sie diese wahrgenommen? 2. Was hätte Ihrer Meinung nach verändert werden können? 3. Was würden Sie sich in ihrer Umgebung wünschen um sich inklusiver behandelt zu fühlen? |  |
| Inklusion in der Gesellschaft | 1. Wie erleben sie die gesellschaftliche Inklusion von Menschen mit Behinderungen? | 1. Welche Massnahmen würden Sie konkret ergreifen, um die Inklusion zu fördern? 2. Welche Barrieren bestehen Ihrer Meinung nach, alle Menschen gleichwertig in der Gesellschaft teilhaben zu lassen? | Können sie noch weitere Beispiele nennen? |
| Rolle der Assistenz-technologie zur Förderung der Inklusion | 1. Welche Bedeutung haben Assistenztechnologien für Menschen mit Behinderungen für Sie? 2. Welche Assistenztechnologien können dabei helfen, die Inklusion von Personen mit einer Behinderung im Arbeitsalltag zu fördern? | 1. Erachten Sie diese als notwendig/wichtig? 2. Welche technischen Hilfsmittel für Personen mit einer körperlichen Behinderung sind Ihnen bekannt? 3. Wie würden Sie Assistenztechnologien steuern wollen? |  |
| Kosten für Assistenz-technologien | 1. Welchen Preis würden Sie für Assistenztechnologien (wie z.B. Roboterarm oder Liegebike) als angemessen erachten? 2. Wie viel der Kosten für assistive Technologie sollte im Arbeitsumfeld vom Arbeitsgeber übernommen werden? | 1. Wie viel der Kosten für einen Assistenztechnologien können selbst getragen werden und wie viel übernimmt die Krankenkasse oder IV? 2. Wie könnte der Staat bei der Finanzierung von Assistenztechnologien unterstützen? |  |
| Abschluss | Gibt es noch weitere Aspekte zum Thema Inklusion, die Sie noch hinzufügen möchten? |  |  |

Bedanken für die Teilnahme, mitteilen, dass die Aufnahme nun gestoppt wird.

Semi-Structured Dialogue on “Inclusion” – Swiss Ability Fair 2024

Introduction and General Information:

Welcome and brief introduction as the moderator

1. Thank participants for taking the time and agreeing to be part of the interview
2. Inform participants about the approximate duration of the interview (about 10 minutes)
3. Mention that the interview will be recorded; recordings will be deleted after data analysis and publication
4. Optionally explain the purpose of the conversation in one or two sentences

Participants' Rights (brief explanation):

1. All data will be treated confidentially, anonymized, and used solely within the context of this project
2. Participation is voluntary
3. You may withdraw at any time without any consequences

Any questions before we begin?

Thank you once again for taking the time to speak with me. I’m now starting the recording.

| **Topic** | **Main question** | **Follow-up-questions** | **Maintaining questions** |
| --- | --- | --- | --- |
| **Introduction** | Could you briefly tell us how old you are and what you do for a living? | 1. What is your gender? 2. What is your profession? 3. How old are you? |  |
| **General Questions** | 1. What does inclusion mean to you? 2. How would you define assistive technologies? | 1. Why do you think inclusion is important in our society? |  |
| **Personal experiences with inclusion** | 1. What personal experiences have you had with inclusion? 2. If so, could you share a positive experience? 3. Have you also encountered challenging or less inclusive situations? | 1. How did you perceive those experiences? 2. What could have been done differently? 3. What would you like to see change in your environment to feel more included? |  |
| **Inclusion in society** | 1. How do you experience the inclusion of people with disabilities in society? | 1. What concrete measures would you take to promote inclusion? 2. What barriers do you see to enabling equal participation for everyone in society? | Can you give additional examples? |
| **Role of assistive technologies in promoting Inclusion** | 1. What importance do assistive technologies have for people with disabilities in your view? 2. What assistive technologies could help promote inclusion in the workplace? | 1. Do you consider them necessary or important? 2. Which assistive devices for people with physical disabilities are you aware of? 3. How would you want to control or interact with assistive technologies? |  |
| **Costs of assistive technologies** | 1. What would you consider a reasonable price for assistive technologies (e.g. robotic arm or recumbent bike)? 2. In a workplace context, how much of the cost should be covered by the employer? | 1. How much of the cost can be covered by individuals themselves, and how much is typically paid by insurance or disability insurance (IV)? 2. How could the state support the financing of assistive technologies? |  |
| **Conclusion** |  | Are there any other aspects related to inclusion that you would like to add? |  |

That brings us to the end of the interview. Thank you very much for your participation. I’m now stopping the recording.
