# Supplementary material for: Inclusion through technology: findings from a public engagement approach
Source: Int J Public Health. 2026 Jul 8;71:1608949. doi: 10.3389/ijph.2026.1608949 (PMC13388228; doi:10.3389/ijph.2026.1608949)
Supplement: Supplementary file 2 [file Supplementaryfile4.pptx]

## Slide 1
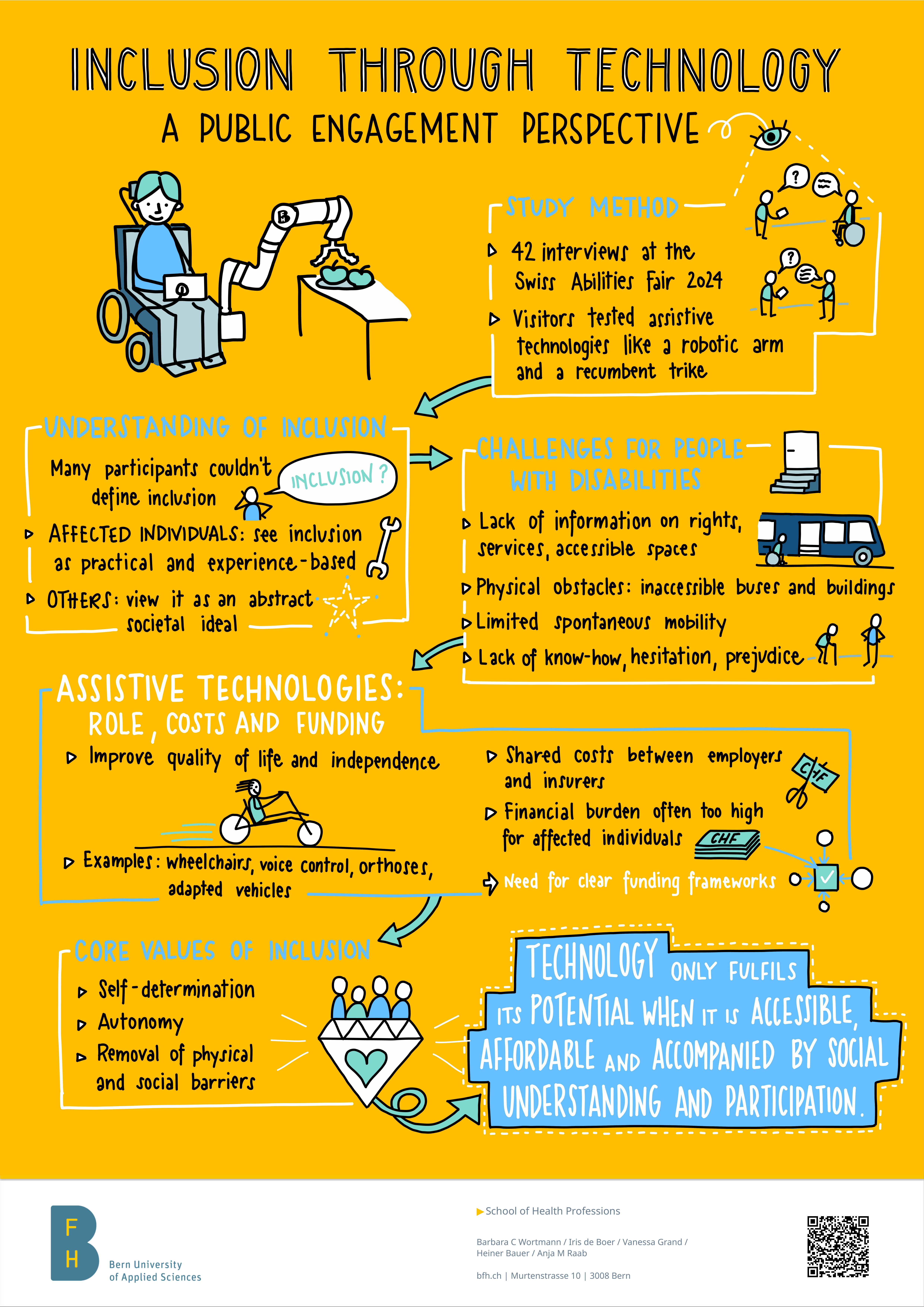

School of Health Professions
# Barbara C Wortmann / Iris de Boer / Vanessa Grand / Heiner Bauer / Anja M Raab bfh.ch | Murtenstrasse 10 | 3008 Bern
