# Supplementary material for: Inclusion through technology: findings from a public engagement approach
Source: Int J Public Health. 2026 Jul 8;71:1608949. doi: 10.3389/ijph.2026.1608949 (PMC13388228; doi:10.3389/ijph.2026.1608949)
Supplement: Supplementary file 3 [file Supplementaryfile2.docx]

| Frage-  blocknr. | Frage 1: Standardfrage BFH-Team | Frage 2: Frage BFH – Team |
| --- | --- | --- |
| 1 | Was verstehen Sie unter Inklusion? | Warum denken Sie ist Inklusion in unserer Gesellschaft wichtig? |
| 2 | Was verstehen Sie unter Inklusion? | Wodurch könnten wir Inklusion in unserer Gesellschaft fördern? |
| 3 | Was verstehen Sie unter Inklusion? | Wie muss eine Assistenztechnologie sein, damit sie inklusiv ist? |
| 4 | Was verstehen Sie unter Inklusion? | Welchen Preis würden Sie für eine Assistenztechnologie (z.B. Roboterarm oder Liegebike) als angemessen erachten? |
| 5 | Was verstehen Sie unter Inklusion? | Wie viel der Kosten für Assistenztechnologien wie z.B. Roboterarm oder Liegebike sollten selbst getragen werden und wie viel übernimmt die Krankenkasse? |
| 6 | Was verstehen Sie unter Inklusion? | Inwiefern trägt Assistenztechnologie dazu bei, eine inklusive Arbeitswelt zu schaffen? |
| 7 | Was verstehen Sie unter Inklusion? | Welche Massnahmen würden Sie konkret ergreifen, um die Inklusion zu fördern? |

**Supplement 2: Short Interview-questions in German and English**

Vorgehen Kurzgespräch:

1. BFH-Team beginnt mit Frage 1. 2. Gesprächspartner: in stellt erste Frage
2. BFH-Team stellt Frage 2. 4. Gesprächspartner: in stellt zweite Frage

Inklusion “einfach” erklärt:

Inklusion bedeutet, dass alle Menschen in der Gesellschaft leben und teilhaben können, unabhängig von allfälligen Unterschieden wie z.B. soz. Hintergrund, Behinderung oder Herkunft). Sie müssen ihr Leben nicht anpassen an die vorhandenen Strukturen – Strukturen werden angepasst, dass alle am Leben teilhaben können.

Aufrechterhaltungsfragen Kurzgespräch:

Allgemein: «Was wäre, wenn Sie selbst in dieser Situation wären?»

Konkrete Beispiele einbringen: «zum Beispiel beim Einkaufen»

Zu Technologie:

« Welche technischen Hilfsmittel kennen Sie denn aus ihrem Alltag?» (Sprachsteuerung, Untertitel…)

«was nervt Sie manchmal an alltäglicher Technologie?»

Zu Inklusion:

« Kennen Sie jemanden in Ihrem Umfeld, der mit Einschränkungen lebt?»

«Haben Sie schon einmal eine Situation erlebt, wo Sie selbst eine Barriere erfahren haben?»

«Ist Ihnen in der Stadt schon einmal etwas aufgefallen, was manchen Menschen Schwierigkeiten macht?»

Wenn keine Antwort kommt: selbst ein Beispiel geben, andere Perspektive anbieten, Multiple Choice anbieten

| Block No. | Question 1: Standard question (Team BFH) | Question 2: Follup-up question (BFH Team) |
| --- | --- | --- |
| 1 | What does inclusion mean to you? | Why do you think inclusion is important in our society? |
| 2 | What does inclusion mean to you? | How could we promote inclusion in our society? |
| 3 | What does inclusion mean to you? | What makes an assistive technology inclusive in your view? |
| 4 | What does inclusion mean to you? | What price would you consider reasonable for an assistive technology (e.g. robotic arm or recumbent bike)? |
| 5 | What does inclusion mean to you? | How much of the cost for assistive technologies such as a robotic arm or recumbent bike should be covered by individuals themselves, and how much should be covered by health insurance? |
| 6 | What does inclusion mean to you? | How do assistive technologies contribute to creating an inclusive work environment? |
| 7 | What does inclusion mean to you? | What specific measures would you take to promote inclusion? |

**Procedure for Short Interview:**

1. The BFH team starts with Question 1 2. The conversation partner then asks their first question.
2. The BFH team continues with Question 2. 4. The conversation partner then asks their second question.

Inclusion – Simple Explanation:

Inclusion means that all people can live and participate in society—regardless of differences such as social background, disability, or origin. People should not have to adapt their lives to fit existing structures—rather, structures must be adapted so that everyone can participate fully in life.

Follow-up / Probing Questions for Short Interview:

General: “What if you were in this situation yourself?”

Concrete examples: “For example, when shopping?”

Regarding technology:

“What technical aids do you know from your daily life?” (e.g. voice control, subtitles…)

“What annoys you about everyday technology?”

Regarding inclusion:

“Do you know anyone in your environment who lives with a disability or limitation?”

“Have you ever experienced a situation where you faced a barrier yourself?”

“Have you ever noticed something in the city that might cause difficulties for some people?”

If there is no answer: Offer your own example, suggest a different perspective, or provide multiple-choice options.
