# Supplementary material for: Inclusion through technology: findings from a public engagement approach
Source: Int J Public Health. 2026 Jul 8;71:1608949. doi: 10.3389/ijph.2026.1608949 (PMC13388228; doi:10.3389/ijph.2026.1608949)
Supplement: Supplementary file 4 [file Supplementaryfile3.pdf]

**Supplement 3: Quotations**

*3-1*

*«Unter Inklusion verstehe ich, dass Menschen mit einer Beeinträchtigung am Gesellschaftsleben können teilnehmen, möglichst uneingeschränkt umfassend.»,*
*Person ohne Gehbeeinträchtigung, Mitte 50, männlich*

*"To me, inclusion means that people with disabilities can participate in social life as fully and with as few restrictions as possible."*
*Male participant without mobility impairment, age mid-50s*

*3-2*

*«Ich verstehe unter Inklusion, dass ich etwas selbstständig machen kann. Dass ich nicht irgendwo jemanden brauche, der mich raufschieben, runtertragen muss. Ja, Eigenständigkeit.»,*
*Person mit Gehbeeinträchtigung, 22, weiblich*

*"To me, inclusion means being able to do something independently. That I don't need someone to push me up or carry me down. Yes, autonomy."*
*Female participant with mobility impairment, age 22*

*3-3*

*«Aber ich finde schon, dass alle Möglichkeiten haben sollten, dass gerade in unserer Gesellschaft, dass alle an unserer Gesellschaft teilhaben können. Ja, einfach am gesellschaftlichen Leben teilhaben. (…) Ich find ziemlich schade, man denkt, dass es nur ein kleiner Teil der Bevölkerung ist. Man denkt dann immer an Menschen mit Beeinträchtigung. Aber hey, Kinderwagen, Leute, die älter sind. Da denkst du jetzt, betrifft dich ja nicht. Aber irgendwann bin ich vielleicht auch mal froh.»*
*Person ohne Gehbeeinträchtigung, 47, weiblich*

*"I do think everyone should have the opportunity to participate in our society. Just to be part of social life. (…) I find it quite unfortunate that people often think it only concerns a small part of the population. They think only of people with disabilities. But hey, strollers, older people—right now you might think it doesn't affect you. But one day, I might be glad too."*
*Female participant without mobility impairment, age 47*

*3-4*

*«Unter Inklusion verstehe ich, dass man unabhängig von jeglicher Voraussetzung, die die Person mitbringt, dass man versucht, alle Leute in die Gesellschaft zu integrieren und vor allem die Voraussetzung schafft, dass sich jeder integriert fühlt und nicht nur fühlt, sondern auch Teilhabe in jeglicher Form haben kann.»*
*Person ohne Gehbeeinträchtigung, 51 Jahre, männlich*

*"To me, inclusion means integrating everyone into society regardless of their individual circumstances, and, above all, creating the conditions that allow everyone not only to feel*

*included, but to truly participate in all aspects of life."*
*Male participant without mobility impairment, age 51*

*3-5*

*«Nur allein, zum Beispiel eben die im Restaurant. Es ist vielleicht, es hat vielleicht breiten Eingang, es steht vorne rollstuhlgerecht, aber die Türen sind eigentlich, ich sag mal, für gewisse Rollstuhlfahrer, die zum WC gehen, gar nicht breit genug sind, viel zu schwer. (…) Oder es steht irgendwo rollstuhlgerecht, aber die Tische sind viel zu niedrig. Oder du kannst gar nicht gescheit runterfahren, dass du gar nicht von dort am Tisch essen könntest, dass du dann quasi auf einem Tablett irgendwie vor dir essen müsstest.»*
*Person ohne Gehbeeinträchtigung., 43, männlich*

*"Take restaurants, for example. The entrance might be wide, and it might say 'wheelchair accessible' at the front—but the doors to the restroom are often too narrow or too heavy for some wheelchair users. (…) Or it says 'accessible,' but the tables are far too low. Or you can't even get close enough to the table to eat properly—you'd have to eat off a tray in front of you."*
*Male participant without mobility impairment, age 43*

*3-6*

*«Wo es dann halt zum Beispiel heisst, ja, ist rollstuhlgängig. Dann kommt man hin und dann geht es eben doch nicht, weil man vielleicht einen zu schweren Rollstuhl hat oder zu breit oder es ist doch eine Stufe da oder sowas.»*
*Person mit Gehbeeinträchtigung, weiblich, Alter unbekannt*

*"Sometimes it says 'wheelchair accessible,' but when you get there, it doesn't actually work—maybe the wheelchair is too heavy or too wide, or there's a step after all."*
*Female participant with mobility impairment, age unknown*

*3-7*

*«Ja, es gibt Gebäude, in die ich wirklich nicht reinkomme, weil es aufgrund der Tetraplegie manchmal schon mühsam ist, Türen aufzutun, die schwer sind oder eben recht unhandlich. Und da muss man halt etwas sein lassen und anders lösen.»*
*Person mit Gehbeeinträchtigung, 25, männlich*

*"There are buildings I simply can't enter because, due to my tetraplegia, it's sometimes really difficult to open heavy or awkward doors. And then you just have to give up and find another solution."*
*Male participant with mobility impairment, age 25*

*3-8*

*«Und ein Rollstuhlfahrer muss halt gezielt aussuchen, wo er den Urlaub verbringen kann. Also voll integriert würde ich heissen, er kann frei drauflosfahren und könnte dann... Frei entscheiden wo er dann bleibt, das kann er definitiv nicht.»*
*Person ohne Gehbeeinträchtigung, 48, männlich*

*"A wheelchair user has to carefully choose where to spend their vacation. Full inclusion would mean being able to travel freely and decide spontaneously where to stay, but that's definitely not possible."*
*Male participant without mobility impairment, age 48*

*3-9*

*«Wenn ich eine Minute zu spät bin, dann gibt es keine Hilfe mehr von der SBB.»*
*Person mit Gehbeeinträchtigung, ca.20, weiblich*

*"If I'm one minute late, there's no more assistance from SBB."*
*Female participant with mobility impairment, age approx. 20*

*3-10*

*«Es ist vielleicht, es hat vielleicht breiten Eingang, es steht vorne rollstuhlgerecht, aber die Türen sind eigentlich, ich sag mal, für gewisse Rollstuhlfahrer, die zum WC gehen, gar nicht breit genug sind, viel zu schwer.»*
*Person mit Gehbeeinträchtigung, 43, männlich*

*"The entrance might be wide, and it might say 'wheelchair accessible' at the front, but the restroom doors are often not wide enough for some wheelchair users and far too heavy to open."*
*Male participant with mobility impairment, age 43*

*3-11*

*«Es gibt viele Hindernisse, wo man halt zum Beispiel als Rollstuhlfahrer gar nicht hin kann. Wo es dann halt zum Beispiel heisst, ja, ist rollstuhlgängig. Dann kommt man hin und dann geht es eben doch nicht, (…) So, Hindernisse halt, wo ich nicht weiterkomme.»*
*Person mit Gehbeeinträchtigung, weiblich, Alter unbekannt*

*There are many obstacles that wheelchair users simply cannot overcome. Sometimes a place is labeled 'wheelchair accessible,' but when you arrive, it turns out not to be (...) These are the kinds of barriers that stop me.", Female participant with mobility impairment, age unknown*

*3-12*

*Sonst bauen sie etwas Neues, zum Beispiel in neuen Häusern, wenn sie einen Lift bauen, mit Touchscreen. Das kann eine blinde Person nicht bedienen.»*
*Person mit Gehbeeinträchtigung, 26, männlich*

*"Sometimes they build something new, like in new buildings, they install elevators with touchscreens. A blind person can't operate that."*
*Male participant with mobility impairment, age 26*

*3-13*

*«Und die sagen, wenn es Leitlinien hat, oder der Bahnhof autonom ist, heisst er ist Rollstuhlgängig, und hat Leitlinien, bieten sie keine Hilfe mehr an. Und das ist halt der Punkt zu*

*sagen, ja klar, ich kann im Rollstuhl alleine fahren, aber ich sehe es nicht. (...) Und auch eine blinde Person, die noch nie an dem Bahnhof war, dann geht das auch nicht. Da muss man sich auch bewusst sein. Dass man sagt, ja, das ist umgebaut. Als blinde Person findet man sich trotzdem nicht zurecht, wenn man den Bahnhof nicht kennt. Da braucht es eine Schulung. Aber man kann nicht sagen, dass man an jedem Bahnhof eine Schulung machen muss, nur weil man einmal dort jetzt umsteigen möchte. Das kann es auch nicht sein.»*
*Person mit Gehbeeinträchtigung, 26, männlich*

*"They say that if a station has tactile guidance lines and is considered wheelchair accessible, no further assistance is provided. But that's the point, yes, I can travel independently in a wheelchair, but I can't see. (…) And for a blind person who has never been to that station, it doesn't work either. People need to be aware of that. Just because a station has been renovated doesn't mean a blind person can find their way around if they don't know it. You'd need training. But you can't expect someone to be trained at every station just because they want to transfer there once. That's not realistic."*
*Male participant with mobility impairment, age 26*

*3-14*

*«Beim Rollstuhl bin ich noch nicht so lange wie blind. Blind ist halt so, ja, dass man viel, sagen wir, in unserer Welt ist halt visuell.»*
*Person mit Gehbeeinträchtigung, 26, männlich*

*"I haven't been using a wheelchair for as long as I've been blind. Being blind is just… well, our world is very visual."*
*Male participant with a mobility impairment, age 26*

*3-15*

*«Bushaltestellen sind zwar inklusiv, aber es hat zum Beispiel keine automatischen Rampen. Da muss der Fahrer kommen, rausklappen, alle nerven sich weil man zu spät kommt und so weiter. Das ist dann genau das: die Probleme machen dann die Menschen dahinter.*
*Person mit Gehbeeinträchtigung, ca. 20, weiblich*

*"Bus stops may be inclusive in theory, but for example, they don't have automatic ramps. The driver has to come out and unfold it manually, and everyone gets annoyed because it causes delays. That's exactly the issue: the problems are caused by the people behind the system. (…) Or when I tell someone I'm taking an English course, the immediate response is, 'Oh, do you need tutoring?' Or the bus driver comments, 'Oh, you're out and about a bit,' when in fact I'm going to school or to work."*
*Female participant with a mobility impairment, age approx. 20*

*3-16*

*«Oder die werden einfach gepackt am Rollstuhl und werden irgendwo hingeschoben.»*
*Person mit Gehbeeinträchtigung, Mitte 50, männlich*

*“Or they’re just grabbed by the wheelchair and pushed somewhere.*
*Male participant without mobility impairment, age mid-50s*

*3-17*

*«…und dann ist natürlich zum Beispiel das iPhone, ohne jetzt Werbung zu machen, es bietet sehr viel für Leute mit einer Sehbehinderung. Unser ältestes Hilfsmittel vielleicht Technologie, Mechanik, ist Rollstuhl oder Gehstock. Oder was hat es noch gegeben? Ein Pflegebett so ganz früher.»*
*Person ohne Gehbeeinträchtigung, 50, weiblich*

*“And then, of course, there’s the iPhone—without wanting to advertise—it offers a lot for people with visual impairments. Our oldest assistive devices, perhaps in terms of technology or mechanics, are the wheelchair or walking stick. Or what else was there? A care bed, way back.”*
*Female participant without mobility impairment, age 50*

*3-18*

*«Das kann vom Gehstock über den Rollstuhl bis zu einem Auto für Rollstuhlgänger sein. Brillen vielleicht auch.»*
*Person ohne Gehbeeinträchtigung, 40, männlich*

*“It can range from a walking stick to a wheelchair, or even a car adapted for wheelchair users. Possibly glasses as well.”*
*Male participant without mobility impairment, age 40*

*3-19*

*«Da kann ich mir ehrlich gesagt gar nicht gross etwas darunter vorstellen. Höchstens, das (...) die Technologie hilft und unterstützt Personen, die etwas brauchen.»*
*Person mit Gehbeeinträchtigung, 27, weiblich*

*“To be honest, I can’t really picture much in that regard. Maybe just that (…) technology helps and supports people who need something.”*
*Female participant with a mobility impairment, age 27*

*3-20*

*«Ich sehe das sehr positiv. Das ist ein Teil der Technik, die man für die Behinderten macht. Wenn ich heute hier sehe, wie viele mit einer Prothese herumlaufen, wenn wir das nicht hätten, hätten wir noch viel mehr Leute, die am Rollstuhl wären (…)»*
*Person ohne Gehbeeinträchtigung, 50, weiblich*

*"I see this very positively. This is a part of technology developed for people with disabilities. When I look around today and see how many people are walking with prostheses—without them, many more would be in wheelchairs.”*
*Female participant with no mobility impairment, age 50*

*3-21*

*«Ich denke, es ist sicher, für Leute mit hohen Einschränkungen sicher förderlich, dass wir solche Dinge haben. Dass wir wirklich selbstständige Sachen machen können, einfach Sachen. Ähm ich sage aber auch, Technik, alles, was man braucht oder nicht braucht, ist eher von Vorteil. Und ja, aber es fördert sicher etwas. Es gibt Sachen, zum Beispiel Lift-Sachen, die schneller gehen, wo man besser in die Gebäude reinkommt oder Türen, die automatisch aufgehen.»*
*Person mit Gehbeeinträchtigung, 25, männlich*

*"I think it's definitely beneficial for people with severe impairments that we have such technologies. They really allow us to do things independently—just simple things. I'd also say that technology, whether you need it or not, is generally an advantage. And yes, it definitely supports something. There are things, like lift systems that work faster, or automatic doors that make it easier to enter buildings."*
*Male participant with mobility impairment, age 25*

*3-22*

*«Was ich sehe, ist, was man wenig hat, ist für die Blinden. Das ist gerade nur ein Stock, den man hat. (…) Ob man da etwas machen könnte mit Sensoren, wo... Ja. Es gibt ja heute wahnsinnig viele Sensoren, wo man alles spürt. Schon wenn man mit dem Auto rückwärtsfährt.»*
*Person ohne Gehbeeinträchtigung, 75, männlich*

*"What I notice is that there's still very little available for people who are blind. It's basically just a cane. (…) Maybe something could be done with sensors… Yes. Nowadays, there are so many sensors that can detect everything—like when you reverse a car."*
*Male participant with no mobility impairment, age 75*

*3-23*

*«Dass man auf seiner Website von einer Firma oder einem Restaurant mehr kommuniziert wird, wie barrierefrei das ist. Weil als jemand mit einem Handicap muss ich noch viel organisieren, das kann man selber auch über das Internet.»*
*Person mit Gehbeeinträchtigung, männlich, Alter unbekannt*

*"It would be helpful if companies or restaurants communicated more clearly on their websites how accessible they are. As someone with a disability, I have to organize a lot in advance—and that's something you could easily do online."*
*Male participant with mobility impairment, age unknown*

*3-24*

*«Also die Leute, die betroffen sind, also von mir ausgesehen, sollten einfach mehr Informationen bekommen, von was sie profitieren können oder wo sie sich melden können, damit man ihnen weiterhelfen kann.»*
*Person ohne Gehbeeinträchtigung, 52, weiblich*

*"From my point of view, people who are affected should simply receive more information, about what support they are entitled to, what they can benefit from, or where they can turn to for help."*
*Female participant with no mobility impairment, age 52*

*3-25*

*«Ich finde die Kommunikation, ob es barrierefrei ist, das sollte mehr da sein. Dass man auf seiner Website von einer Firma oder einem Restaurant mehr kommuniziert wird, wie barrierefrei das ist. Weil als jemand mit einem Handicap muss ich noch viel organisieren, das kann man selber auch über das Internet.»*
*Person mit Gehbeeinträchtigung, 47, männlich*

*"I believe there should be more communication about accessibility, especially on the websites of companies or restaurants. As someone with a disability, I have to organize a lot in advance, and that kind of information could easily be provided online."*
*Male participant with mobility impairment, age 47*

*3-26*

*«Wobei ich merke, dort ist die Hürde noch recht gross. Das ist oft recht schwierig. (…) die Leute, Trainer, die dort sind, die haben das Know-how nicht, die haben vielleicht Angst, dass sie das nicht genau umsetzen können, sie haben ähm vielleicht auch Möglichkeiten nicht, um die Rahmenbedingungen anzupassen, (…) oder vielleicht gewisse Vorurteile gegenüber mit ähm irgendwelchen Behinderungen, so Sachen.»*
*Person ohne Gehbeeinträchtigung, 51, männlich*

*""I notice that the barriers are still quite high in this area. It's often quite difficult. (…) The people involved, like trainers, often lack the necessary know-how. They may be afraid of not being able to implement things properly, or they simply don't have the means to adapt the conditions (...) There may also be certain prejudices toward people with disabilities."*
*Male participant with no mobility impairment, age 51*

*3-27*

*«Wenn wir zusammen raften gehen, du zahlst 50 Stutz, ich zahle 300 Stutz, ist das nicht fair. Also da ist irgendwo ein Punkt, wo ich der Meinung bin, es muss... Gleichberechtigung heisst auch Gleichberechtigung in Zahlen.»*
*Person mit Gehbeeinträchtigung, 39, männlich*

*"When we go rafting together, you pay 50 francs, I pay 300—that's not fair. At some point, I think we need to say: equality also means equality in numbers."*
*Male participant with mobility impairment, age 39*

*3-28*

*«(…) die Tochter hat eine CP und sie war Kindergarten bis Zweite in einer öffentlichen Schule. Dann begannen die Lehrer zu bocken. (...) Wir fanden, das gehe nicht mehr, auch mit der*

*Assistenz nicht.»*
*Person ohne Gehbeeinträchtigung, 52, weiblich*

*"(…) Our daughter has CP and attended a public school from kindergarten through second grade. Then the teachers started to resist. (…) We felt it was no longer working, even with the support of an assistant."*
*Female participant with no mobility impairment, age 52*

*3-29*

*«Also gerade bei den kleinen Kindern, wo es darum geht, Start in Kindergarten oder Kinderkrippe oder so, da sehe ich wie zwei Welten oder Parallelen. Also so Krippe geht oft noch sehr gut, dass Kind inklusive Kinder mit Zerebralparese zum Beispiel. Ich sehe auch, dass die Leute sehr engagiert sind. Also auch Leute, die gar nichts mit dem Thema zu tun haben, sind eigentlich berührt und wollen helfen und versuchen, dass es geht und oft ist das dort auch finanziell noch möglich. Die Hilfe ist weniger, wenn das Kind klein, also es ist weniger aufwendig oder streng körperlich zum Helfen. Die Leute sind sehr interessiert dort. Also man versucht wirklich. Und dann Kindergarten, Schulen fehlen oft die Ressourcen. (…) Also mehr Personal, mehr Finanzen, aber auch Räume. Ich glaube auch, viel Verständnis, das fehlt.»*
*Person ohne Gehbeeinträchtigung, 45, weiblich*

*"Especially with young children, when it comes to starting kindergarten or daycare, I see two very different worlds. Daycare often works quite well, even for children with cerebral palsy, for example. I notice that people are very committed. Even those who have no prior connection to the topic are often touched and want to help, and they try to make it work. Financially, it’s often still manageable at that stage. Supporting small children is also less physically demanding. People are genuinely interested. But once children enter kindergarten or school, resources are often lacking, more staff, more funding, but also space. And I think there’s also a lack of understanding."*
*Female participant with no mobility impairment, age 45*

*3-30*

*«Da gehen Kinder in ein anderes Dorf in die Sonderschule oder in die heilpädagogische Schule. Und dann die sind wie weg aus dem Dorf. Die sind gar nicht mehr so dabei. Das finde ich mega schade. Ich finde, man sollte schon bei den kleinen Kindern anfangen. (…) Ja, also ich meine, ich finde, dass meine Kinder, die haben gar nie Kontakt mit Kindern mit einer Beeinträchtigung.»*
*Person ohne Gehbeeinträchtigung, 44, weiblich*

*"Children are sent to special schools or schools for children with special needs in another village. And then they’re just gone from the community. They’re no longer really part of it. I think that’s such a shame. We should start with the youngest children. (…) I mean, my own children have never had any contact with children with disabilities."*
*Female participant with no mobility impairment, age 44*

*3-31*

*«Er ist ganz normal in der Regelklasse vom Kindergarten. Wo wir neu dazugekommen sind, ist eigentlich extra eine Rampe gemacht worden für den Eingang, dass er selber rein kann und selber die Tür aufmachen kann, weil das andere ist so nicht möglich. Und er kann ganz normal dabei sein.»*
*Person ohne Gehbeeinträchtigung, Mitte 30, männlich*

*"He attends a regular kindergarten class. When we joined, a ramp was specifically built at the entrance so he could enter independently and open the door himself, otherwise, it wouldn't have been possible. Now he can fully participate like everyone else."*
*Male participant with no mobility impairment, age mid-30s*

*3-32*

*«(…), weil wenn man sich bewirbt an einer Stelle und es ist schon klar, dass der Arbeitgeber da was investieren muss, dann nimmt der einen vielleicht deswegen nicht.»*
*Person mit Gehbeeinträchtigung, weiblich, Alter unbekannt*

*"(…) because when you apply for a job and it's already clear that the employer would have to invest in accommodations, they might decide not to hire you because of that."*
*Female participant with mobility impairment, age unknown*

*3-33*

*«Ich bin aber auch der Meinung, mit dem ganzen Homeoffice-Thema, dass man sehr viele von den Umbaumassnahmen umgehen kann und Leute im Homeoffice kann schaffen lassen, was für viele viel besser ist. Ich (...) schaffe von zu Hause aus, aber ich kann eine 2-Stunden-Mittagspause machen Ich kann mich während der zwei Stunden hinlegen, meine Beine entlasten. Wenn ich Schmerzen habe, kann ich das Stehpult reinstellen, kann den Laptop mit rübernehmen. Das sind so die Sachen, die es für mich viel einfacher machen von Zuhause zu arbeiten als an irgendeinem Arbeitsplatz etwas zu finden, was gut ist.»,*
*Person mit Gehbeeinträchtigung, 39, männlich*

*"I also believe that with the whole remote work topic, many of the costly workplace modifications can be avoided. Letting people work from home is often a much better solution. I (...) work from home, and I can take a two-hour lunch break, lie down, and relieve my legs. When I'm in pain, I can set up a standing desk or move my laptop around. These are the things that make working from home much easier for me than trying to find a suitable setup at a conventional workplace."*
*Male participant with mobility impairment, age 39*

*3-34*

*«Ich denke, man müsste versuchen, eine Art 50-50 Lösung hinbekommen. Also man sagt der Versicherung die Hälfte und die Hälfte der Arbeitgeber.»*
*Person mit Gehbeeinträchtigung, 69, männlich*

*"I think we should aim for a kind of 50-50 solution, where half of the costs are covered by the insurance and the other half by the employer."*
*Male participant with mobility impairment, age 69$*

*3-35*

*«Das bedeutet auch Freiheit und Selbstständigkeit.»*
*Person mit Gehbeeinträchtigung, 22, weiblich*

*“It also means freedom and independence.”*
*Female participant with mobility impairment, age 22*

*3-36*

*«Sehr grosse, das ist eine wertvolle Sache sicher. Ich bin froh gibt es den Rollstuhl, ich bin froh gibt es Orthesen. Ohne das könnte ich im Alltag nicht so teilnehmen, wie ich es jetzt machen kann.»*
*Person mit Gehbeeinträchtigung, 27, weiblich*

*“Very much so, it’s definitely something valuable. I’m grateful for the wheelchair, I’m grateful for the orthoses. Without them, I wouldn’t be able to participate in everyday life the way I do now.”*
*Female participant with mobility impairment, age 27*

*3-37*

*Ich denke, sehr grosse Bedeutung, insbesondere würde ich meinen, sollte man mit Hilfsmitteln versuchen, möglichst viele Situationen zu lösen, aber dort natürlich auch die Hilfsmittel möglichst einfach zu bedienen, im Sinne von Sprachkommunikation, weil es sagt, wenn ich... Ich bin so hochgelähmt, dass ich nichts ohne Weiteres mit den Händen etwas machen kann.»*
*Person mit Gehbeeinträchtigung, 69, männlich*

*“I think it’s very important. In my view, assistive devices should be used to solve as many situations as possible, but they also need to be easy to operate, especially through voice control. I say this because I’m so highly paralyzed that I can’t easily use my hands for anything.”*
*Male participant with mobility impairment, age 69*

*3-38*

*«Ich finde, es sollte immer ein guter Rahmen sein und es ist halt die Frage, ob man selber bezahlen muss oder nicht. Weil selber haben wir meistens als Behinderte nicht Mittel quasi, um das selber zu finanzieren.»*
*Person mit Gehbeeinträchtigung, 22, weiblich*

*“I think there should always be a solid framework, but the question is whether you have to pay for it yourself or not. As people with disabilities, we usually don’t have the financial means to cover these costs ourselves.”*
*Female participant with mobility impairment, age 22*

*3-39*

*«Die meisten Menschen mit Behinderungen leben ja am Existenzminimum und haben keine Reserven. Deshalb find ich es einfach frech, wenn sie so Kosten selber übernehmen würden. Ich*

*meine, sie können nichts für diese Behinderung und sie sind so eingeschränkt und deshalb sollte man es ihnen wenigstens ermöglich, dass Hilfsmittel übernommen werden. Weil ich meine, ein normaler Mensch hätte diese Kosten auch nicht. Das kann nicht sein. Ich meine, ich z.B. mit meiner Behinderung, ich muss so viel Kosten tragen, nur, dass ich atmen kann.»*
*Person mit Gehbeeinträchtigung, 22, weiblich*

*"Most people with disabilities live at the subsistence level and have no financial reserves. That's why I find it outrageous to expect them to cover such costs themselves. I mean, they're not responsible for their disability, and they're already so limited, at the very least, assistive devices should be covered. A non-disabled person wouldn't have these expenses. It's just not right. For example, with my disability, I have to bear so many costs just to be able to breathe."*
*Female participant with mobility impairment, age 22*

*3-40*

*«Ich finde es generell gut, ähnlich wie bei der Krankenversicherung, wenn der Nutzer auch einen Anteil trägt. Das muss aber angepasst sein an die finanziellen Verhältnisse von den Nutzern.»*
*Person ohne Gehbeeinträchtigung, Mitte 40, männlich*

*"In general, I think it's a good idea, similar to health insurance, if users also contribute a share. But it has to be adjusted to their financial situation."*
*Male participant without mobility impairment, mid-40s*

*3-41*

*«Und darum finde ich auch, dass es gewisse Sachen gibt, die Gesellschaft tragen sollte, um den Einzelnen zu unterstützen.»*
*Person ohne Gehbeeinträchtigung, Mitte 30, männlich*

*"That's why I also believe there are certain things society should cover, to support the individual."*
*Male participant without mobility impairment, mid-30s*

*3-42*

*«Solange es einem Patienten das Leben, ich sage jetzt nicht erleichtert, aber ermöglicht, finde ich es eigentlich von der Krankenkasse oder auch der IV übernommen werden. Wenn es irgendwelche Sachen sind, wo man sagt, sage jetzt nicht einfach das Luxusgut ist, sondern das Leben vielleicht einfacher macht, sei das das so ein kleiner Roboter, der nachfährt zum Einpacken oder so finde ich, ist dann vielleicht eine anteilige Beteiligung angebracht. Und das was also wirklich solche extra Sachen sind, wo er einfach will, weil er es cool findet, sollte er auch selber müssen zahlen.»*
*Person ohne Gehbeeinträchtigung, Mitte 30, männlich*

*"As long as something enables a person to live, not just makes life easier, I believe it should be covered by health insurance or disability insurance. If it's something that simply makes life more convenient, like a small robot that helps with packing, then partial coverage might be appropriate.*

*But for extras someone just wants because they think it's cool, they should pay for it themselves."*
*Male participant without mobility impairment, mid-30s*

*3-43*

*«Hilfsmittel so wie es notwendig ist. Luxushilfsmittel dann halt eher privat»*
*Person mit Gehbeeinträchtigung, ca. 20, weiblich*

*"Assistive devices should be provided as needed. Luxury aids, however, should be privately financed."*
*Female participant with mobility impairment, approx. age 20*

*3-44*

*«Aber wenn ich mir etwas wünschen könnte, dann wäre es schon, dass der Selbstbehalt vielleicht nicht bei 10-20% liegt und nicht höher, weil dann habt ihr noch alle Zugang zu Hilfsmitteln.»*
*Person ohne Gehbeeinträchtigung, 30, weiblich*

*"If I could make a wish, it would be that the co-payment stays at around 10–20%, not higher, so that everyone still has access to assistive devices."*
*Female participant without mobility impairment, age 30*

*3-45*

*«Aber das ist natürlich eine Herausforderung, weil man nicht so einfach so günstig wird. Aber wenn man auch Forschung betreibt, Sachen günstiger zu machen in der Entwicklung, und zu Stückzahlen kommen, die höher sind und vielleicht auch noch Fördermöglichkeiten findet in anderen Ländern, dann kann man die Preise schon noch deutlich senken.»*
*Person ohne Gehbeeinträchtigung, Mitte 40, männlich*

*"Of course, that's a challenge, because it's hard to make things affordable. But if research focuses on reducing development costs, increasing production volumes, and finding funding opportunities in other countries, prices could be significantly lowered."*
*Male participant without mobility impairment, mid-40s*

*3-46*

*«Ich meine, eine elektrische Tür hilft auch einem Fussgänger oder einem Kellner. (…) Ich glaube ich meine, ich merke nur, früher mit dem Kinderwagen, oder? Und jetzt kann man einfach reinfahren. Es sind so kleine... Und das dient allen eigentlich.»*
*Person ohne Gehbeeinträchtigung, 50, weiblich*
*"An automatic door helps not only wheelchair users, it also benefits pedestrians or waiters. (…) I noticed it myself when I had a stroller. Now you can just roll in. These small things serve everyone."*
*Female participant without mobility impairment, age 50*
